# Characteristics Associated with Multimorbidity among Older United States Adult Opioid Users with Pain

**DOI:** 10.3390/jcm12206684

**Published:** 2023-10-23

**Authors:** David R. Axon, Megan Grieser

**Affiliations:** 1 Department of Pharmacy Practice & Science, R. Ken Coit College of Pharmacy, University of Arizona, 1295 N Martin Ave., P.O. Box 210202, Tucson, AZ 85721, USA; megangrieser@arizona.edu; 2 Center for Health Outcomes and PharmacoEconomic Research (HOPE Center), R. Ken Coit College of Pharmacy, University of Arizona, 1295 N Martin Ave., P.O. Box 210202, Tucson, AZ 85721, USA

**Keywords:** multimorbidity, opioids, pain

## Abstract

The objective of this study was to investigate the variables associated with multimorbidity status among older United States (US) adults with self-reported pain and opioid use. This study used a cross-sectional retrospective database design that included US adults aged ≥50 years with self-reported pain who used an opioid in 2019 in the Medical Expenditure Panel Survey data. Multivariable logistic regression models, weighted to produce nationally representative estimates, were used to determine variables significantly associated with multimorbidity status (≥2 versus <2 chronic conditions). Significance was determined using an a priori alpha level of 0.05. In the adjusted logistic regression analysis, those aged 50–64 (vs. ≥65 years), Hispanic (vs. non-Hispanic), employed (vs. unemployed), and who performed frequent exercise (vs. no frequent exercise) were associated with lower odds of having multimorbidity. In conclusion, these characteristics may be targets for pain management and opioid use interventions among older US adults. Further research is needed to investigate the variables associated with multimorbidity in greater detail.

## 1. Introduction

There is an ongoing opioid crisis in the United States (US) [1]. In 2018, approximately 32% of US adults received a prescription for opioids within the last two years [1]. Addiction, respiratory depression, and physical dependence are common side effects of long-term opioid use, with four million people reporting nonmedical use of opioids and 2357 opioid-related overdose deaths in 2022 [2,3]. Opioids are a particular problem among older adults, with findings from one study revealing 2.2% of adults aged 65 and above had used prescription opioids for non-medical purposes within the past year, and 5% had done so at some point in their lives [4]. An opioid is an analgesic that acts on three opioid receptors (DOP, KOP, MOP), either as an agonist, partial agonist, or antagonist. Clinically relevant opioids will act as agonists and bind to the MOP receptors within the peripheral nervous system to elicit analgesia. Compounds within this class include morphine, codeine, thebaine, papaverine, diamorphine (heroin), dihydromorphine, buprenorphine, oxycodone, pethidine, fentanyl, methadone, alfentanil, remifentanil, and tapentadol [5]. Opioids are typically effective at managing pain and are commonly prescribed for pain management as a standard of care throughout the world [6,7]. From 2003 through 2017, an average of 8.2 billion morphine milligram equivalents (MMEs) were prescribed by 669,495 prescribers to 3.9 million patients each year in the US [8]. Total healthcare expenditures are up to 105% greater among individuals prescribed an opioid compared against those not prescribed an opioid [9].

The International Association for the Study of Pain (IASP) revised the definition of pain to an unpleasant sensory and emotional experience associated with, or resembling that associated with, actual or potential tissue damage [10]. However, pain is subjective, and many other definitions also exist. Despite pain being one of the most common medical complaints, many patients still suffer from untreated or undertreated pain [11]. Approximately 100 million adults (or roughly 30% of adults) suffer from pain, with a misdiagnosis rate of up to 40–80% for chronic pain patients [12,13]. Pain can negatively impact quality of life when left untreated and can progress from an acute to a chronic condition, which results in considerable morbidity and mortality [14].

Alongside pain, many older US adults also have multimorbidity [15], which is defined as the coexistence of two or more chronic conditions in the same person [16]. The prevalence of those with multimorbidity continues to rise in the US [17], with approximately 81% of US adults 65 years and older and 50% of US adults aged 45 to 65 years having multimorbidity [18]. Like pain, multimorbidity contributes to poorer health and quality of life [15].

The proportion of older adults is increasing both in the US and globally due to an increasing life expectancy [19]. It is estimated that one-third of older adults with multimorbidity take analgesics regularly [20]. There has also been research showing an association between multimorbidity and individual factors that may precipitate this status [21]. To this end, there is a need to investigate the variables associated with multimorbidity among older US adults with pain. There is also an interest specifically in assessing multimorbidity among older adults who use opioids. Therefore, the objective of this study was to assess the variables associated with multimorbidity status among older US adults with pain interference in the past four weeks who used opioids.

## 2. Methods

### 2.1. Study Design, MEPS, and Study Eligibility

This study applied a cross-sectional retrospective design that utilized 2019 Medical Expenditure Panel Survey (MEPS) data. The MEPS comprises substantial surveys questioning individual people, their families, their medical providers, and their employers throughout the US. The MEPS collects information on health service consumption by Americans, including the frequency of service use, cost of service use, and payment methods for service use. The MEPS also contains comprehensive information about the breadth and cost of health insurance utilized by Americans. The MEPS has three principal constituents: the Household component, the Insurance component, and the Medical Provider component. The Household component was utilized in this study, which pertains to data from all members in a residence. These data may be supplemented with that from healthcare providers in the Medical Provider component. The Household component collects data from all members in a residence obtained from a selection of residences included in the sampling framework from the previous year’s National Health Interview Survey (NHIS). During interviews, characteristics that are collected include demographics, health conditions and status, medical service utilization, payment charges and sources, healthcare access, healthcare satisfaction, healthcare coverage, employment information, and income. These data are collected telephonically over two calendar years [22]. The 2019 full-year consolidated data file (HC-216) utilized for this study contains data for 28,512 individuals and is publicly accessible to researchers [23,24]. Study subjects had to be alive for the entire 2019 calendar year in the US, be aged 50 years and older, have self-reported pain interference in the previous four weeks, and have reported using at least one opioid in 2019. Pain was identified using the following item: during the past four weeks, how much did pain interfere with your normal work (including work outside the home and housework)? This item had response options of not at all, little bit, moderately, quite a bit, and extremely. In this study, an individual was determined to have pain if they responded with anything other than not at all. Use of at least one opioid was identified in the 2019 MEPS prescribed medicines file (HC-213A) using Multum Lexicon therapeutic class codes for opioids (code 60) and combination opioids (code 191) [25,26].

### 2.2. Variables

The independent variables were organized using a modification of the Anderson behavioral model, which comprised predisposing variables, enabling variables, need variables, personal health practice variables, and external environmental variables [27]. Predisposing variables comprised age, sex, race, and ethnicity. Enabling variables comprised education completed, employment status, marital status, and income level. Need variables comprised any limitation, pain severity, general health status, and mental health status. Personal health practice variables comprised frequent exercise status and smoking status. External environmental variables comprised the census region. 

The dependent variable was multimorbidity status. Multimorbidity was defined as having at least two conditions from this subsequent list of conditions that were available in the MEPS full-year consolidated data file: angina, coronary heart disease, hypertension, other heart disease/condition, myocardial infarction, stroke, hypercholesterolemia, diabetes, asthma, chronic bronchitis, emphysema, arthritis, joint pain, and cancer. The comparison was no multimorbidity, which included individuals with zero or one condition from the above list. 

### 2.3. Data Analysis

Differences in variables between the multimorbidity and no multimorbidity groups were compared using chi-squared tests. The percentage of each comorbid condition was summarized as a weighted percentage with 95% confidence interval (CI). The characteristics associated with multimorbidity versus no multimorbidity were assessed using an adjusted logistic regression model, which included all the variables described above. Variables were added iteratively, and the model with the best c-statistic was used (in this case, the model with all the variables produced the best c-statistic). Statistical significance was indicated using an alpha level of 0.05 established a priori. Variables provided within the MEPS data set were used to maintain clusters and strata within data and to compute nationally representative estimates of the population. A secondary sub-group analysis was conducted, stratifying the data by sex to determine if the findings differed by sex. An adjusted logistic regression model similar to that described above was developed for each sex. All analyses were conducted using SAS (v9.4, SAS institute Inc., Cary, NC, USA). This study was approved by the University of Arizona Institutional Review Board (protocol number #00001403, 20 June 2022). 

## 3. Results

**Figure 1:**
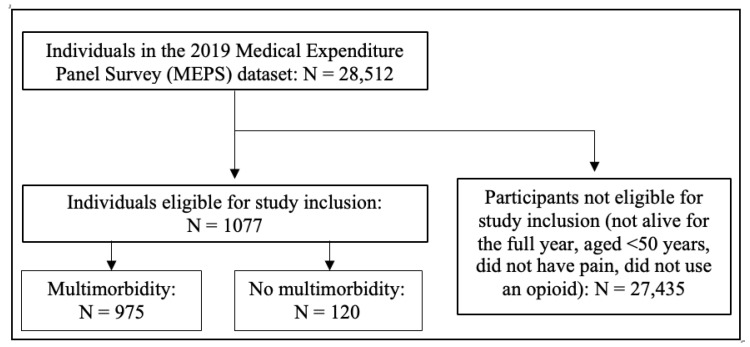
Study eligibility flowchart.

Among the available sample of 28,512 individuals in the 2019 MEPS data set, 1077 met the eligibility criteria and were included in this study. There were 957 individuals with multimorbidity and 120 individuals with no multimorbidity (Figure 1). This represented a weighted population of 12,109,702 individuals, of which 10,671,315 (88.1%, 95% CI = 85.8, 90.4) had multimorbidity and 1,438,387 (11.9%, 95% CI = 9.6, 14.2) had no multimorbidity. 

The majority of older US adults with pain interference in the past four weeks who used an opioid in this study were aged over 65 years old, female, white, non-Hispanic, more than high school educated, unemployed, married, had a middle–high income, had a limitation, had little/moderate pain, had excellent/very good/good perceived physical health, had excellent/very good/good perceived mental health, did not frequently exercise, and did not smoke. The most common census region was the South, then the Midwest, West, and Northeast. Statistical differences existed among the groups for the following groups: age 50–64 versus ≥65, Hispanic versus non-Hispanic ethnicity, employed versus unemployed, any limitation (yes versus no), excellent/very good/good versus fair/poor perceived physical health, and frequent exercise (yes versus no) (Table 1). 

**Figure 2:**
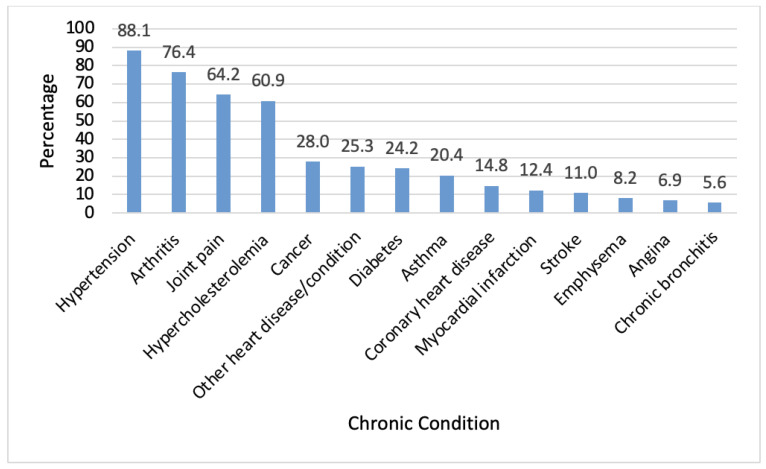
Weighted percentage of each chronic condition present among older United States adults (age ≥ 50 years) with self-reported pain interference in the past four weeks who used opioids.

The most common chronic conditions reported were hypertension (88.1%), followed by arthritis (76.4%), joint pain (64.2%), and hypercholesterolemia (60.9%). Less common conditions included cancer (28.0%), other heart diseases (25.3%), diabetes (24.2%), and asthma (20.4%). The remaining conditions were reported by less than 15% of the population. (Figure 2).

In the adjusted logistic regression analysis, the following variables had lower odds of being associated with multimorbidity in the full cohort of people: age 50–64 versus ≥65 years, Hispanic versus non-Hispanic ethnicity, employed versus unemployed, and frequent exercise (yes versus no). There was no statistical association between the remaining variables and multimorbidity (Table 2). 

In the sub-group analysis stratified by sex, age 50–64 versus ≥65 years and Hispanic versus non-Hispanic ethnicity still had lower odds of being associated with multimorbidity among females, but not among males. Meanwhile, being employed versus unemployed still had lower odds of being associated with multimorbidity among males but not among females. Despite no association in the overall cohort, being married versus other marital status had higher odds of being associated with multimorbidity among males but not among females. Conversely, there was no statistical association between frequent exercise status among males or females, despite an association being observed in the overall cohort (Table 3). 

## 4. Discussion

The key findings from this study were that among the total cohort studied, those who were aged 50–64 (versus ≥65 years), Hispanic (versus non-Hispanic) ethnicity, employed (versus unemployed), and doing frequent exercise (yes versus no) were each associated with lower odds of multimorbidity in the adjusted logistic regression analysis. However, some differences were observed between sexes in the secondary sub-group analysis. These findings are discussed in greater detail below. 

The first variable associated with multimorbidity status among older US opioid users with pain interference in the past four weeks was age. Those aged 50–64 years old (versus ≥65 years old) were associated with lower odds of having multimorbidity in the full cohort analysis and the female subgroup analysis, but not in the male subgroup analysis. Put another way, females aged ≥65 years old (versus 50–64 years old) were associated with higher odds of having multimorbidity. This finding adds new knowledge to fill a gap in the literature about the association of age and multimorbidity status among older US opioid users with pain. In a previous study, adults with prescription opioid misuse were shown to be older (over 50 years old) US adults who suffered from poor quality of life, suggesting the need for multidisciplinary help with these patients [28]. The literature indicates about 12.0% of US adults aged 45–64 and 4.4% of adults aged 25–44 experience chronic and high-impact pain, compared to 10.7% of US adults aged 65–84 and 15.8% of those over 85 years old [29]. This suggests there may be a need to expand our work beyond the current study to assess pain management for those aged 25–44 and those over 85 years. Research has found that pain is underreported and unmanaged in the aging US population, for instance, 66% of residents at one geriatric nursing home reported having chronic pain, but 34% of these cases were undetected by the treating physician [30]. Pain may be influenced by multimorbidity status in the geriatric population, as one study found a correlation between multimorbidity status, chronic pain, and age over 80 years among community-dwelling Mexican Americans [31]. 

The second variable associated with multimorbidity status among older US opioid users with pain interference in the past four weeks was ethnicity. Those of Hispanic ethnicity (versus non-Hispanic ethnicity) were associated with lower odds of multimorbidity in the full cohort analysis and the female subgroup analysis, but not in the male subgroup analysis. A recent study found that non-Hispanic whites received opioids more than other ethnic groups at emergency department visits [32]. However, other evidence suggests that the Latino community is facing an increasing risk of opioid abuse, as demonstrated in high-risk counties in California [33]. The National Institutes of Health have reported that culture is a strong influence on how Hispanic Americans experience pain, and while they may be more sensitive to pain, they report fewer pain conditions such as back pain [34].

The third variable associated with multimorbidity status among older US opioid users with pain interference in the past four weeks was employment. Those who were employed (versus unemployed) had lower odds of multimorbidity in the full cohort analysis and the male subgroup analysis, but not in the female subgroup analysis. Alternatively, this finding shows that males who were unemployed were associated with greater odds of multimorbidity. It is perhaps likely that those with greater multimorbidity (that may be due to pain contributing to their pain) are less able or unable to work due to their multiple health conditions. Work disability has been found to be as common as 1 in 10 working US adults aged 18–65 years old, which may (at least partially) explain the findings of the current study [35]. Previous research has shown that chronic disease is more prevalent among those who are unemployed, including such physiological disorders as cardiovascular disease and diabetes. Pain has been implicated with a decline in employment in the American workforce over the past several decades: 43% of working-aged men are not employed due to a serious health condition, with half of these men reporting daily use of painkillers [36]. Work disability is associated with fewer employment opportunities, poorer economic outcomes, and a greater reliance on social programs, resulting in low employment [37]. Further research in this area could be insightful in exploring the impact of employment on multimorbidity among older US adults with pain who use opioids. 

The fourth variable associated with multimorbidity status among older US opioid users with pain interference in the past four weeks was exercise. Those who reported doing frequent physical exercise, in this study defined as at least 30 min of moderate to high-intensity physical exercise at least five times a week (versus no frequent physical exercise), were associated with lower odds of having multimorbidity in the full cohort analysis. This may be considered a logical finding since those who have multimorbidity may be less able or unable to frequently exercise. A recent study also using MEPS data found that just over one-third of US opioid users with pain over the age of 50 considered themselves frequent exercisers (as defined in the current study), and that age, health status, weight, and pain severity were associated with frequent exercise [38]. This contrasts with just over half (50.3%) of US adults, regardless of pain or opioid use status, in another MEPS study [39]. A meta-analysis found that there was an inverse relationship between physical activity and multimorbidity in a population of 77,000 older US adults [40]. Available evidence indicates that pain severity can be improved through frequent physical exercise [41]. This finding may be influenced by a healthy user bias, defined by the National Institutes of Health as the propensity for patients who receive one preventative therapy to take action to engage in other behaviors that also improve health [42]. This finding is important in the role that it can play in the guidance of non-pharmaceutical pain management recommendations. For instance, exercise has been reported as a pain management strategy used by community-dwelling adults, pharmacists, and students, to give just some examples [43,44]. However, the association between exercise status and multimorbidity was not observed when the males and females were analyzed separately. Further research is warranted to examine sex-based differences in the association between exercise and multimorbidity, specifically among the population of older US adults with pain who use opioids. While it is well known that frequent physical exercise is beneficial for health, further research in this area could be helpful to determine causality and to suggest healthcare interventions or lifestyle modifications to improve health outcomes, specifically for the population of older US opioid users with pain. 

Finally, being married versus other marital status had higher odds of being associated with multimorbidity in the male subgroup analysis. This was not the case among the female subgroup of the overall cohort. There is limited literature to explain this finding. Previous research around the influence of marital status on health outcomes for various conditions has been conducted, such as in one study on women with breast cancer [45]. Other studies have looked at marital status and comorbid conditions after cardiovascular surgery [46], while others have looked at marital support and mental health [47]. From a healthcare utilization perspective, previous research has shown that married Medicare beneficiaries had higher odds of outpatient healthcare utilization and lower odds of an inpatient stay or skilled nursing facility stay [48]. The current study demonstrates an association between marital status and multimorbidity among US older males who have pain and use opioids. Additional research to establish the relationship between marital status, sex, and comorbidities may be helpful to investigate this topic further. 

The strengths of this study include the large, nationally representative sample of US adults with pain who use opioids, which should indicate good external validity for this population. Limitations of this study include the inability to establish a cause-and-effect or temporal relationship due to the cross-sectional retrospective design of the study and recall bias from the self-reported responses to the MEPS questions. Another limitation is the subjective nature of pain and the varied definitions that exist, making it difficult to compare the current study’s findings to existing literature. One final limitation is that opioid use in this study included any opioid use in 2019, which may have been a one-off or acute dose of a single opioid or could represent frequent or chronic opioid use or one or multiple opioids. In addition, the type of opioid and the route of administration were not known, which may cause variation in the findings. Future research may be warranted to disaggregate this analysis by other demographic characteristics to further explore any differences that may exist among these groups of people. 

## 5. Conclusions

The findings of this study suggest variables (age, ethnicity, employment, exercise) that may be the most optimal targets for healthcare interventions or lifestyle modifications to reduce the prevalence of multimorbidity among older (≥50 years) US opioid users with pain, which currently stands at 88.1% of this population. Further research is required to establish a temporal relationship between these variables and multimorbidity in this population to better explain these findings. 

## Figures and Tables

**Table 1:** Characteristics of United States older adults (age ≥ 50 years) with self-reported pain interference in the past four weeks who used opioids stratified by multimorbidity status.

Variables	Total (N = 1077) Weighted % (95% Confidence Interval)	Multimorbidity ≥2 Chronic Conditions (N = 957) Weighted % (95% Confidence Interval)	No Multimorbidity <2 Chronic Conditions (N = 120) Weighted % (95% Confidence Interval)	*p*
Predisposing variables				
Age (years)				<0.0001
50–64	48.0 (44.3, 51.7)	45.5 (41.7, 49.3)	66.9 (57.5, 76.2)	
≥65	52.0 (48.3, 55.7)	54.5 (50.7, 58.3)	33.1 (23.8, 42.5)	
Sex				0.6522
Male	39.7 (36.6, 42.7)	39.4 (36.1, 42.6)	41.8 (31.6, 52.0)	
Female	60.3 (57.3, 63.4)	60.6 (57.4, 63.9)	58.2 (48.0, 68.4)	
Race				0.1117
White	82.6 (79.5, 85.8)	81.8 (78.3, 85.3)	88.6 (82.1, 95.1)	
Other	17.4 (14.2, 20.5)	18.2 (14.7, 21.7)	11.4 (4.9, 17.9)	
Ethnicity				0.0314
Hispanic	7.3 (5.4, 9.2)	6.6 (4.6, 8.6)	12.4 (6.3, 18.5)	
Non-Hispanic	92.7 (90.8, 94.6)	93.4 (91.4, 95.4)	87.6 (81.5, 93.7)	
Enabling variables				
Education completed				0.0755
High school or less	48.0 (44.3, 51.6)	49.1 (45.3, 53.0)	39.1 (28.8, 49.4)	
More than high school	52.0 (48.4, 55.7)	50.9 (47.0, 54.7)	60.9 (50.6, 71.2)	
Employment status				<0.0001
Employed	31.3 (27.9, 34.6)	28.5 (25.2, 31.9)	51.6 (40.9, 62.2)	
Unemployed	68.7 (65.4, 72.1)	71.5 (68.1, 74.8)	48.4 (37.8, 59.1)	
Marital status				0.7195
Married	54.2 (50.5, 58.0)	54.5 (50.5, 58.4)	52.5 (41.8, 63.1)	
Other	45.8 (42.0, 49.5)	45.5 (41.6, 49.5)	47.5 (36.9, 58.2)	
Income level				0.9360
Poor/near poor/low income	35.9 (32.5, 39.3)	35.9 (32.4, 39.4)	35.5 (25.7, 45.3)	
Middle/high income	64.1 (60.7, 67.5)	64.1 (60.6, 67.6)	64.5 (54.7, 74.3)	
Need variables				
Any limitation				0.0354
Yes	68.2 (64.6, 71.8)	69.6 (65.8, 73.3)	58.2 (47.5, 69.0)	
No	31.8 (28.2, 35.4)	30.4 (26.7, 34.2)	41.8 (31.0, 52.5)	
Pain severity				0.2366
Little/moderate	54.8 (51.2, 58.4)	54.0 (50.3, 57.8)	60.8 (50.1, 71.5)	
Quite a bit/extreme	45.2 (41.6, 48.8)	46.0 (42.2, 49.7)	39.2 (28.5, 49.9)	
General health status				0.0073
Excellent/very good/good	60.8 (57.4, 64.1)	59.0 (55.5, 62.5)	74.1 (64.5, 83.6)	
Fair/poor	39.2 (35.9, 42.6)	41.0 (37.5, 44.5)	25.9 (16.4, 35.5)	
Mental health status				0.0691
Excellent/very good/good	79.9 (77.1, 82.7)	79.0 (76.1, 81.9)	86.5 (79.6, 93.4)	
Fair/poor	20.1 (17.3, 22.9)	21.0 (18.1, 23.9)	13.5 (6.6, 20.4)	
Personal health practice variables				
Frequent exercise				0.0033
Yes	37.8 (34.0, 41.6)	35.8 (31.7, 39.9)	53.2 (42.2, 64.2)	
No	62.2 (58.4, 66.0)	64.2 (60.1, 68.3)	46.8 (35.8, 57.8)	
Smoking status				0.3734
Yes	23.7 (21.0, 26.4)	23.2 (20.4, 26.0)	27.3 (18.2, 36.5)	
No	76.3 (73.6, 79.0)	76.8 (74.0, 79.6)	72.7 (63.5, 81.8)	
External environmental variables				
Census region				0.3390
Northeast	12.7 (8.7, 16.8)	12.5 (8.7, 16.2)	14.7 (5.6, 23.7)	
Midwest	24.4 (20.8, 28.0)	24.3 (20.4, 28.1)	25.0 (15.7, 34.3)	
South	39.6 (35.2, 44.0)	40.7 (36.1, 45.2)	31.5 (21.6, 41.4)	
West	23.3 (20.0, 26.6)	22.6 (18.8, 26.3)	28.9 (19.9, 37.8)	

**Table 2:** Characteristics associated with multimorbidity (versus no multimorbidity) among United States older adults (age ≥ 50 years) with self-reported pain interference in the past four weeks who used opioids.

Variables	Adjusted Odds Ratio (95% Confidence Limits)
Predisposing variables	
Age 50–64 vs. ≥65 years	**0.472 (0.292, 0.763)**
Male vs. female sex	1.031 (0.653, 1.626)
White vs. other race	0.601 (0.290, 1.247)
Hispanic vs. non-Hispanic ethnicity	**0.460 (0.228, 0.929)**
Enabling variables	
High school or less vs. higher than high school education	1.415 (0.807, 2.480)
Employed vs. unemployed	**0.516 (0.292, 0.913)**
Married vs. other marital status	1.139 (0.697, 1.861)
Poor/near poor/low income vs. middle/high income	0.702 (0.415, 1.185)
Need variables	
Any limitation: yes vs. no	1.121 (0.618, 2.034)
Little/moderate vs. quite a bit/extreme pain severity	1.234 (0.721, 2.111)
Excellent/very good/good vs. fair/poor general health status	0.550 (0.299, 1.010)
Excellent/very good/good vs. fair/poor mental health status	0.823 (0.395, 1.715)
Personal health practice variables	
Frequent exercise: yes vs. no	**0.552 (0.327, 0.932)**
Smoker: yes vs. no	0.657 (0.399, 1.081)
External environmental variables	
Northeast vs. West census region	1.212 (0.596, 2.463)
Midwest vs. West census region	1.200 (0.619, 2.326)
South vs. West census region	1.423 (0.788, 2.569)

Analysis based on an unweighted sample n = 1077 (multimorbidity (≥2 chronic conditions) n = 957; no multimorbidity (<2 chronic conditions) n = 120) United States adults alive during the calendar year 2019, aged ≥50 years, with self-reported pain in the past four weeks who used opioids. The reference group in the binomial logistic regression models had no multimorbidity (<2 chronic conditions). The model had a c-statistic of 0.715 and a Wald value of *p* = 0.0001. Bold indicates the characteristic has a significant association with multimorbidity (≥2 chronic conditions).

**Table 3:** Characteristics associated with multimorbidity (versus no multimorbidity) among United States older adults (age ≥ 50 years) with self-reported pain interference in the past four weeks who used opioids, stratified by male and female sex subgroups.

Variables	Male Adjusted Odds Ratio (95% Confidence Limits)	Female Adjusted Odds Ratio (95% Confidence Limits)
Predisposing variables		
Age 50–64 vs. ≥65 years	0.442 (0.194, 1.004)	**0.503 (0.262, 0.967)**
White vs. other race	0.405 (0.120, 1.367)	0.645 (0.247, 1.683)
Hispanic vs. non-Hispanic ethnicity	0.703 (0.210, 1.349)	**0.353 (0.149, 0.834)**
Enabling variables		
High school or less vs. higher than high school education	1.041 (0.472, 2.297)	1.868 (0.950, 3.672)
Employed vs. unemployed	**0.339 (0.145, 0.789)**	0.662 (0.309, 1.418)
Married vs. other marital status	**2.049 (1.003, 4.185)**	0.863 (0.433, 1.718)
Poor/near poor/low income vs. middle/high income	0.906 (0.362, 2.268)	0.565 (0.285, 1.120)
Need variables		
Any limitation: yes vs. no	0.750 (0.324, 1.735)	1.588 (0.806, 3.129)
Little/moderate vs. quite a bit/extreme pain severity	1.126 (0.481, 2.635)	1.179 (0.555, 2.508)
Excellent/very good/good vs. fair/poor general health status	0.861 (0.382, 1.941)	0.327 (0.160, 1.665)
Excellent/very good/good vs. fair/poor mental health status	0.739 (0.236, 2.315)	1.151 (0.481, 2.755)
Personal health practice variables		
Frequent exercise: yes vs. no	0.628 (0.266, 1.480)	0.576 (0.301, 1.110)
Smoker: yes vs. no	0.701 (0.339, 1.453)	0.666 (0.339, 1.309)
External environmental variables		
Northeast vs. West census region	0.519 (0.170, 1.588)	2.459 (0.717, 8.433)
Midwest vs. West census region	0.892 (0.335, 2.375)	1.289 (0.605, 2.743)
South vs. West census region	0.970 (0.375, 2.509)	1.541 (0.743, 3.198)

Analysis based on an unweighted sample n = 1077 (multimorbidity (≥2 chronic conditions) n = 957; no multimorbidity (<2 chronic conditions) n = 120) United States adults alive during the calendar year 2019, aged ≥50 years, with self-reported pain in the past four weeks who used opioids. The reference group in the binomial logistic regression models had no multimorbidity (<2 chronic conditions). The male sex model had a c-statistic of 0.732, and the female sex model had a c-statistic of 0.737. Bold indicates the characteristic has a significant association with multimorbidity (≥2 chronic conditions).

## Data Availability

Data are available from the corresponding author upon reasonable request.
